# Supplementary material for: Older adults select different but not simpler strategies than younger adults in risky choice
Source: PLoS Comput Biol. 2024 Jun 10;20(6):e1012204. doi: 10.1371/journal.pcbi.1012204 (PMC11192436; doi:10.1371/journal.pcbi.1012204)
Supplement: S1 Text — (PDF) [file pcbi.1012204.s001.pdf]

Risk profiles of the strategies

To examine whether the different distributions of strategies can capture the age differences in risk aversion, we simulated the choices of the five most frequently selected strategies on the choice problems in Pachur, Mata, & Hertwig [1]. Averaging across 100 repeated simulations, we computed the risk aversion of each strategy separately for gain, loss, and mixed problems (Figure S1.1). The strategies that were more frequently selected by older adults according to the resource-rational strategy selection model (Maximax, Equal weight) showed lower risk aversion in gain and mixed problems than the strategies that were more frequently selected by younger adults according to the model (Minimax, Least likely, Priority heuristic). This pattern is consistent with the empirical observation that older adults were less risk averse than younger adults in gain and mixed problems, but not in loss problems.

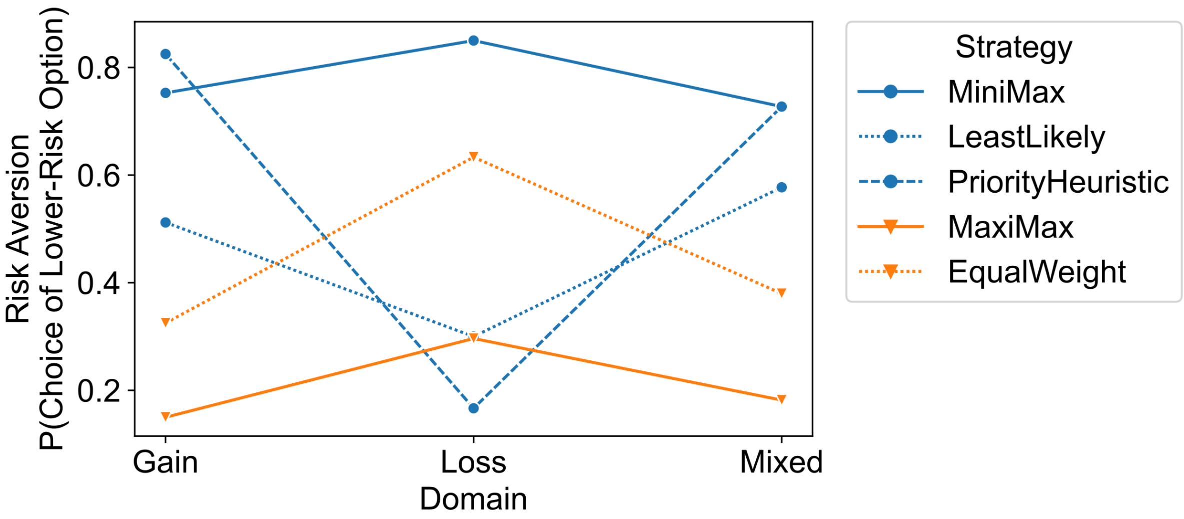

Figure S1.1

Simulated risk aversion for the five strategies that were estimated by the resource-rational strategy selection model to be selected most frequently. Blue lines with circles indicate the strategies that were more frequently selected by younger adults and orange lines with triangles indicate the strategies that were more frequently selected by older adults.

References

[1] Pachur T, Mata R, Hertwig R. Who Dares, Who Errs? Disentangling Cognitive and Motivational Roots of Age Differences in Decisions under Risk. Psychological Science. 2017;28(4):504–518. doi:10.1177/0956797616687729.
