# Supplementary material for: Older adults select different but not simpler strategies than younger adults in risky choice
Source: PLoS Comput Biol. 2024 Jun 10;20(6):e1012204. doi: 10.1371/journal.pcbi.1012204 (PMC11192436; doi:10.1371/journal.pcbi.1012204)
Supplement: S2 Text — (PDF) [file pcbi.1012204.s002.pdf]

Are age differences in strategy selection mediated by age differences in affect?

To further examine the viability of a motivational account of age differences in risky choice, we assessed to what extent the effect of age was mediated by differences in positive and negative affect. In the study by Pachur, Mata, & Hertwig [1], positive and negative affect were assessed with a short version of the Positive Affect Negative Affect Schedule–Extended (PANAS-X; [2]). Two older adults, for whom these variables were not recorded, were not included in this analysis. As reported in [1], the older adults reported lower negative affect and higher positive affect than the younger adults (which is a typical finding, see [2, 3]).

To compute the total effect of age group on model-predicted risk aversion, we used mixed-effects beta regression to predict the proportion of choices of the option with the lower risk according to the simulations of the fitted resource-rational strategy selection model, using age group (younger vs. older), problem domain (gain, loss, and mixed), and their interaction as fixed effects, and with random intercepts for participants and choice problems. Except from the slightly lower sample size, this analysis was identical to the posterior predictive check reported in the main text. To compute the direct (i.e., unmediated) effect of age group on model-predicted risk aversion, we fitted an extended regression model that additionally included positive affect, the interaction of positive affect and problem domain, negative affect, and the interaction of negative affect and problem domain. The indirect (i.e., mediated) effect of age group on model-predicted risk aversion was computed by subtracting the direct effect from the total effect. To quantify the strength of the mediation, we computed the ratio of the indirect effect over the total effect P_M [4].

Table S2.1 shows the total, direct, and indirect effect of age groups as well as the effects of positive affect and negative affect on model-predicted risk aversion, separately for gain and loss problems (the two domains for which age differences were observed). Although the credible intervals for the indirect effects did not exclude 0, the P_M ratios suggest that up to a third of the effect of age group on model-predicted risk aversion

was mediated by positive and negative affect (gain problems: $P_M = 0.33$, mixed problems: $P_M = 0.31$).

Table S2.1

Results of the mediation analysis testing to what extent the age differences in risk aversion as modeled with the resource-rationality strategy selection model are mediated by positive and negative affect.

	Gain problems		Mixed problems	
Effect	b	95% CI	b	95% CI
Age group (total effect)	−0.12	[−0.20, −0.03]	−0.12	[−0.21, −0.03]
Age group (direct effect)	−0.08	[−0.18, 0.02]	−0.08	[−0.18, 0.02]
Age group (indirect effect)	−0.04	[−0.17, 0.10]	−0.04	[−0.17, 0.10]
Positive affect	−0.01	[−0.06, 0.04]	−0.02	[−0.07, 0.04]
Negative affect	0.04	[−0.02, 0.10]	0.04	[−0.02, 0.10]

References

[1] Pachur T, Mata R, Hertwig R. Who Dares, Who Errs? Disentangling Cognitive and Motivational Roots of Age Differences in Decisions under Risk. Psychological Science. 2017;28(4):504–518. doi:10.1177/0956797616687729.

[2] Grühn D, Kotter-Grühn D, Röcke C. Discrete Affects across the Adult Lifespan: Evidence for Multidimensionality and Multidirectionality of Affective Experiences in Young, Middle-Aged and Older Adults. Journal of Research in Personality. 2010;44(4):492–500. doi:10.1016/j.jrp.2010.06.003.

[3] Kunzmann U, Little TD, Smith J. Is Age-Related Stability of Subjective Well-Being a Paradox? Cross-sectional and Longitudinal Evidence from the Berlin Aging Study. Psychology and Aging. 2000;15(3):511–526. doi:10.1037/0882-7974.15.3.511.

[4] Shrout PE, Bolger N. Mediation in Experimental and Nonexperimental Studies: New Procedures and Recommendations. Psychological Methods. 2002;7(4):422–445. doi:10.1037/1082-989X.7.4.422.
