# Supplementary material for: Older adults select different but not simpler strategies than younger adults in risky choice
Source: PLoS Comput Biol. 2024 Jun 10;20(6):e1012204. doi: 10.1371/journal.pcbi.1012204 (PMC11192436; doi:10.1371/journal.pcbi.1012204)
Supplement: S3 Text — (PDF) [file pcbi.1012204.s003.pdf]

Application of the resource-rational strategy selection model to another dataset

To examine the generalizability of our conclusions for the dataset by Pachur, Mata, & Hertwig [1], we applied the resource-rational strategy selection model to the dataset collected by Horn, Schaltegger, Best, & Freund [2], who also examined risky choices in younger and older adults. To our knowledge, this is the only other dataset available for which there is a relatively large number of choices per participant, which is a prerequisite for reliably estimating the model parameters. It should be noted, however, that Horn et al. [2] did not find age differences in decision quality and risk aversion (a likely reason for this discrepancy with regard to the findings by Pachur et al. [1] is that their sample of older adults had considerably higher education levels). In that sense, the datasets by Horn et al. [2,] and Pachur et al. [1,] are somewhat difficult to compare. Still, given that similar decision behavior might be achieved by younger and older adults using different strategies (e.g., [3]), the dataset by Horn et al. [2] might still be an informative testbed for the current purposes.

Horn et al. [2] had collected risky choices from 147 younger adults (71 female, 68 male, 8 other, mean age: 25.4 years, range: 18–36 years) and 139 older adults (79 female, 60 male, mean age: 70.2 years, range: 60–89 years), recruited via Prolific Academic. Participants completed 78 risky choice problems, consisting of two gambles each with two outcomes and corresponding probabilities. Choice problems were evenly distributed across the gain, loss, and mixed domain. The type of incentivization was manipulated between participants; half of the participants received a financial bonus based on one randomly drawn choice and the other half received a financial bonus based on all their choices. Because there were no differences in decision quality and risk aversion between incentivization conditions, we collapsed both conditions for the purpose of our current analysis.

Posterior Predictive Checks

For each participant, we computed the proportion of trials in which the choice predicted by the model matched the empirically observed choice (averaging across 100

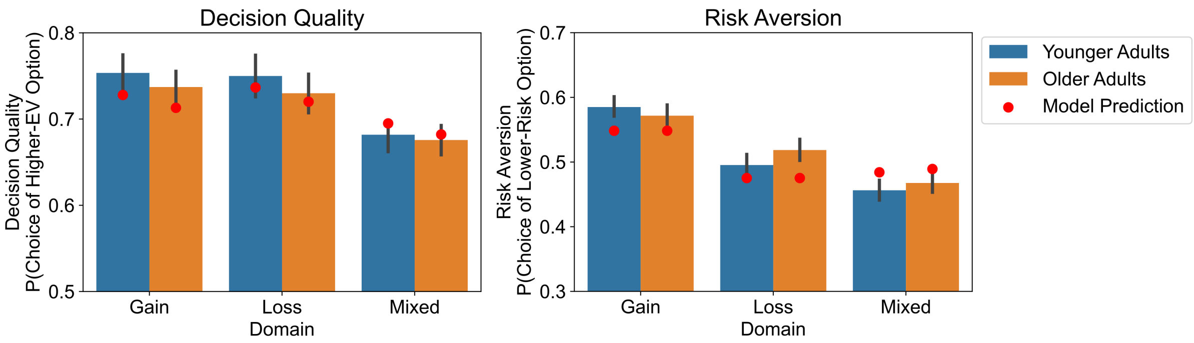

Figure S3.1

Decision quality (left) and risk aversion (right) by problem domain and age group in the dataset by Horn et al. [2]. Bars show the empirically observed behavior (error bars represent the standard error of the mean), red dots show the average predictions of the resource-rational strategy selection model.

model simulations). The proportion of matching choices was, on average, 0.70 in younger adults (range: 0.50–0.95) and 0.67 in older adults (range: 0.51–0.84). A two-sided Bayesian t-test indicated moderate evidence that the match of the simulated with the empirical choices differed between age groups ($BF_{10} = 3.6$). In other words, the resource-rational strategy selection model seemed to capture the choices of younger adults slightly better than those of older adults.

Figure S3.1 shows the decision quality and risk aversion as empirically observed and as predicted by the resource-rational strategy selection model. Since in the Horn et al. [2] dataset the age differences in decision quality and risk aversion were not statistically significant, we did not conduct a statistical analysis of age differences in the model predictions. Note, however, that the model described the overall behavioral patterns in the data quite well.

We also compared for each participant their empirically observed decision quality and risk aversion (pooled across all three problem domains) against the decision quality and risk aversion of the choices simulated with their best-fitting model parameters (Figure S3.2). The higher a participant's decision quality, the higher was the decision quality of their simulated choices ($r = 0.94, BF_{10} = 1.0 \times 10^{127}$). Moreover, the simulated levels of risk aversion were strongly correlated with the empirical ones

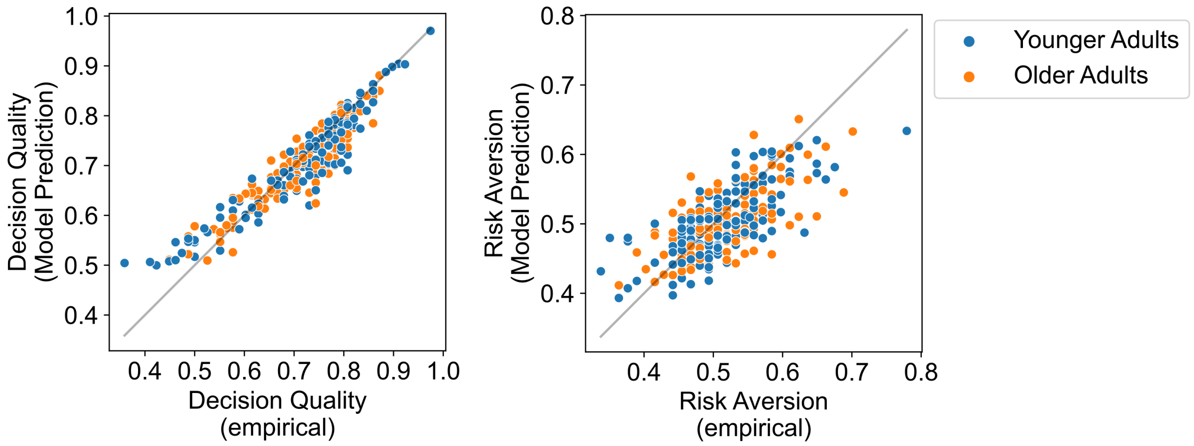

Figure S3.2

Comparison of simulated (based on the fitted resource-rational strategy selection model) and empirically observed levels of decision quality (left) and risk aversion (right) in the dataset by Horn et al. [2]. Each circle represents one participant (with the choices pooled across the three domains; i.e., gain, loss, mixed). The diagonal lines indicate identity (i.e., perfect fit).

($r = 0.69, BF_{10} = 6.1 \times 10^{37}$). This analysis shows that similar as for the data by Pachur et al. [1] reported in the main text, the resource-rational strategy selection model captured the individual differences in choice behavior between participants rather well.

Age Differences in the Model Parameters

Although younger and older adults did not differ in decision quality and risk aversion, they might still rely on different strategies. A Bayesian contingency-table test showed that there was strong evidence that the distribution of strategies differed between age groups ($BF_{10} = 2.8 \times 10^{47}$, Cramér's $V = .11$; Figure S3.3). The strategies with the more pronounced age differences in strategy use were the least-likely strategy (more often selected by older adults) and the equal-weight strategy (more often selected by younger adults).

To compare model parameters between younger and older adults, we conducted one-sided Bayesian t-tests. There was moderate evidence that older adults did not incur lower costs by the strategies used ($BF_{10} = 0.11$), moderate evidence that older adults did not have smaller strategy toolboxes ($BF_{10} = 0.19$), strong evidence that the

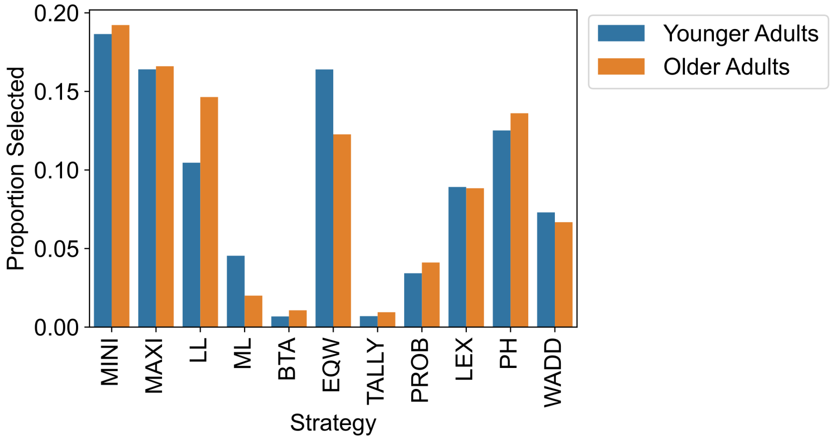

Figure S3.3

Frequency of strategies across all trials in the dataset by Horn et al. [2]. MINI: Minimax, MAXI: Maximax, LL: Least-likely, ML: Most-likely, BTA: Better-than-average, EQW: Equal-weight, TALLY: Tallying, PROB: Probable, LEX: Lexicographic, PH: Priority heuristic, WADD: Weighted-additive.

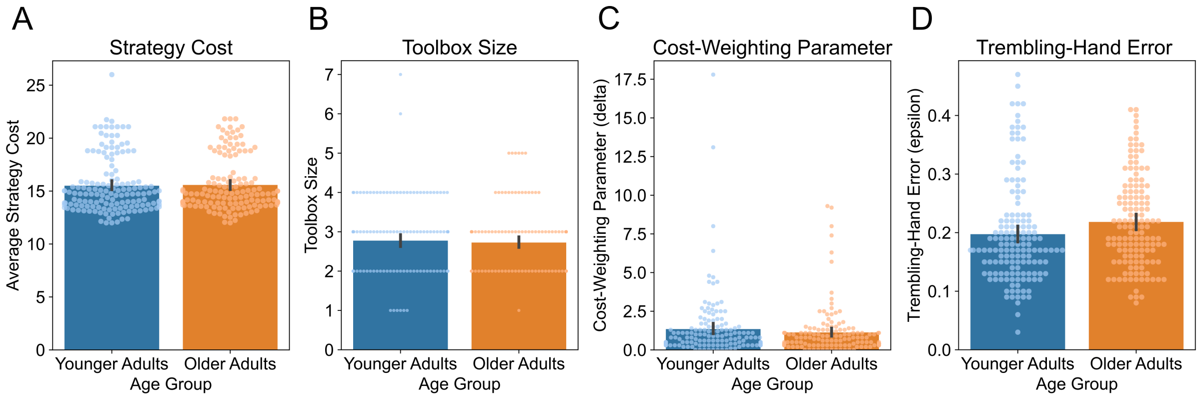

Figure S3.4

Average strategy cost (A), toolbox size (B), cost-weighting parameter (C), and trembling-hand error (D) for younger and older adults in the dataset by Horn et al. [2]. Bars indicate group means, with error bars representing the standard error of the mean. Points represent individual participants.

cost-weighting parameter was not larger for older adults ($BF_{10} = 0.07$), and inconclusive evidence that the trembling-hand error is higher for older adults ($BF_{10} = 2.3$). Overall, these findings corroborate the conclusion from the main text that age differences in strategy selection are not predominantly driven by cognitive factors.

Parameter Recovery Analysis

We conducted a parameter recovery analysis similar to the analysis conducted for the choice problems from Pachur et al. [1] also for the Horn et al. [2] data. For the cost-weighting parameter delta, the recovered parameter values were correlated with the data-generating parameters at $r = .35$. For the trembling-hand error, the recovered parameter values were correlated with the data-generating parameter values at $r = .90$. The recovered toolbox sizes were correlated with the data-generating toolbox sizes at $r = .50$. When computing the average strategy cost for each agent, we found a correlation of $r = .47$ between the cost of the data-generating and the recovered strategies. On average, the data-generating strategy was recovered correctly in 53.7% of trials. For comparison, a model that guesses an agent's strategies would correctly recover the strategies in only 9.1% of trials.

Model Comparison

When comparing the model fit between the resource-rational strategy selection model and cumulative prospect theory, cumulative prospect theory captured the choice data better than the resource-rational strategy selection model for both younger and older adults (Table S3.1).

Table S3.1

Log-likelihoods of the resource-rational strategy selection model and cumulative prospect theory for the dataset by Horn et al. [2]. Higher values indicate a better fit.

Model	Full sample	Younger adults	Older adults
Resource-rational strategy selection	-10926	-5434	-5492
Cumulative prospect theory	-10712	-5330	-5382

References

[1] Pachur T, Mata R, Hertwig R. Who Dares, Who Errs? Disentangling Cognitive and Motivational Roots of Age Differences in Decisions under Risk. Psychological Science. 2017;28(4):504–518. doi:10.1177/0956797616687729.

[2] Horn S, Schaltegger T, Best R, Freund AM. Pay One or Pay All? The Role of Incentive Schemes in Decision Making across Adulthood. The Journals of Gerontology: Series B. 2023;78(1):51–61. doi:10.1093/geronb/gbac108.

[3] Li Y, Baldassi M, Johnson EJ, Weber EU. Complementary Cognitive Capabilities, Economic Decision Making, and Aging. Psychology and Aging. 2013;28(3):595–613. doi:10.1037/a0034172.
