# Supplementary material for: Older adults select different but not simpler strategies than younger adults in risky choice
Source: PLoS Comput Biol. 2024 Jun 10;20(6):e1012204. doi: 10.1371/journal.pcbi.1012204 (PMC11192436; doi:10.1371/journal.pcbi.1012204)
Supplement: S4 Text — (PDF) [file pcbi.1012204.s004.pdf]

Comparison of the strategies' decision profiles

To analyze the extent to which the strategies considered in our analysis have distinguishable decision profiles, we simulated the decisions of each strategy on the risky choice problems of Pachur, Mata, & Hertwig [1] 100 times. For each pair of strategies, we computed the percentage of problems for which both strategies predicted the same choice. For most of the strategies (8 out of 11), the overlap between their decision profiles was less than 77%. Three strategies (Most-likely, Probable, Lexicographic) showed highly similar decision profiles (more than 92% overlap). However, since in our modeling analysis none of these strategies was estimated to be selected frequently by participants of either age group, the effect of potential confusion between these three strategies should be negligible.

References

[1] Pachur T, Mata R, Hertwig R. Who Dares, Who Errs? Disentangling Cognitive and Motivational Roots of Age Differences in Decisions under Risk. Psychological Science. 2017;28(4):504–518. doi:10.1177/0956797616687729.
