# Supplementary material for: Older adults select different but not simpler strategies than younger adults in risky choice
Source: PLoS Comput Biol. 2024 Jun 10;20(6):e1012204. doi: 10.1371/journal.pcbi.1012204 (PMC11192436; doi:10.1371/journal.pcbi.1012204)
Supplement: S5 Text — (PDF) [file pcbi.1012204.s005.pdf]

Specification of strategy cost

Each strategy was specified as a chain of required elementary information processes (EIP; cf. [1]). As in previous work, we used the following set of EIPs: reading, comparing, difference, adding, product, eliminating, moving, and choosing. For some strategies, the number of EIPs varied across choice problems; in these cases, we indicate the range of possible EIPs. The specifications below do not necessarily reflect the temporal sequence of the EIPs (e.g., for the minimax strategy, comparing the outcomes of the first option is already possible before all outcomes of all options have been read).

Minimax

Move to and read all outcomes ($n = 8$). Compare the second outcome of an option to the first outcome of the same option to identify the option's minimum outcome ($n = 2$). Compare the minimum outcomes of each option ($n = 1$) and choose the option with the higher minimum outcome ($n = 1$).

Maximax

Move to and read all outcomes ($n = 8$). Compare the outcome to the current maximum outcome ($n = 3$). Choose the option with the higher maximum outcome ($n = 1$).

Least-likely

Move to and read all outcomes ($n = 8$). Compare the outcomes of each option to identify the option's minimum outcome ($n = 2$). Move to the minimum outcome of each option, move to the corresponding probability, and read the probability ($n = 6$). Compare the probabilities of the minimum outcome of each option ($n = 1$), and choose the option with the lower probability of the minimum outcome ($n = 1$).

Most-likely

Move to and read all probabilities ($n = 8$). Compare the probabilities of each option to identify the option's highest probability ($n = 2$). Move to the highest probability of each option, move to the corresponding outcome, and read the outcome ($n = 6$). Compare the most likely outcomes of each option ($n = 1$) and choose the

option whose most likely outcome is higher ($n = 1$).

Better-than-average

Move to and read all outcomes ($n = 8$). Compare each outcome to the grand average of the outcomes ($n = 4$); if the outcome is higher than the grand average, add 1 to the number of better-than-average outcomes of the option ($n \in \{1, 2, 3\}$). Compare the number of better-than-average outcomes between the two options ($n = 1$) and choose the option with the higher number of better-than-average outcomes ($n = 1$).

Equal-weight

Move to and read all outcomes ($n = 8$). Add the outcome to current sum of outcomes ($n = 4$). Compare the sum of outcomes between the two options ($n = 1$) and choose the option with the higher sum of outcomes ($n = 1$).

Tallying

Move to and read all outcomes ($n = 8$). Compare the outcomes of an option to identify the option's minimum and maximum outcomes ($n = 2$). For each option, move to the minimum outcome, move to the corresponding probability, and read the probability ($n = 6$). For each option, move to the maximum outcome, move to the corresponding probability, and read the probability ($n = 6$). Compare the minimum outcome between the two options ($n = 1$) and add a tallymark to the option with the higher minimum outcome ($n \in \{1, 2\}$). Compare the maximum outcomes between the two options ($n = 1$) and add a tallymark to the option with the higher maximum outcome ($n \in \{1, 2\}$). Compare the probabilities of the minimum outcomes between the two options ($n = 1$) and add a tallymark to the option with the lower probability for the minimum outcome ($n \in \{1, 2\}$). Compare the probabilities of the maximum outcomes between the two options ($n = 1$) and add a tallymark to the option with the higher probability of the maximum outcome ($n \in \{1, 2\}$). Compare the tallymarks between the options and choose the option with the higher number of tallymarks ($n = 2$)

Probable

Move to and read all probabilities ($n = 8$). Compare each probability to the threshold (when there are two outcomes, the threshold is 0.5; $n = 4$). If the probability is probable (i.e., equal or higher than the threshold), move to the outcome, read the outcome, and add it to the current sum of outcomes ($n \in \{6, 9, 12\}$). If the number of probable outcomes for an option is larger than 1, divide the sum of outcomes by the number of outcomes ($n \in \{0, 1, 2\}$). Compare the mean of probable outcomes between the options ($n = 1$) and choose the option with the higher mean of probable outcomes ($n = 1$).

Lexicographic

Move to and read all probabilities ($n = 8$). Compare all probabilities of an option to identify the option's highest probability ($n = 2$). Move to the highest probability of each option, move to the corresponding outcome, and read the outcome ($n = 6$). Compare the most likely outcomes of each option ($n = 1$). If both outcomes are equal, eliminate the two outcomes ($n \in \{0, 2\}$), move to and read all remaining outcomes ($n \in \{0, 4\}$), and then compare the outcomes ($n \in \{0, 1\}$). Choose the option with the higher outcome on the relevant comparison ($n = 1$).

Priority heuristic

Using a gain problem as an example: Move to and read all outcomes ($n = 8$). Compare outcomes of an option to identify the option's minimum outcome ($n = 2$). Determine the difference between the minimum outcomes of the options and compare the difference to the threshold (which is 10% of the maximum outcome in a choice problems; $n = 2$). If the difference is below the threshold, move to the minimum outcome of each option, move to the corresponding probability, and read the probability ($n \in \{0, 6\}$), then take the difference of the probabilities and compare it to the threshold (which is .1; $n \in \{0, 2\}$). If the difference is below the threshold, eliminate the minimum outcomes from the options ($n \in \{0, 2\}$), move to and read the remaining outcomes ($n \in \{0, 4\}$), and compare the outcomes ($n \in \{0, 1\}$). Choose the option with the more attractive value on the respective attribute ($n = 1$).

Weighted-additive

Move to and read all outcomes and probabilities ($n = 16$). Multiply each outcome with its corresponding probability ($n = 4$) and add to the option's probability-weighted outcomes ($n = 4$). Compare the weighted sums ($n = 1$) and choose the option with the higher weighted sum ($n = 1$).

References

[1] Johnson EJ, Payne JW. Effort and Accuracy in Choice. Management Science. 1985;31(4):395–414. doi:10.1287/mnsc.31.4.395.
