# Supplementary material for: Older adults select different but not simpler strategies than younger adults in risky choice
Source: PLoS Comput Biol. 2024 Jun 10;20(6):e1012204. doi: 10.1371/journal.pcbi.1012204 (PMC11192436; doi:10.1371/journal.pcbi.1012204)
Supplement: S6 Text — (PDF) [file pcbi.1012204.s006.pdf]

Preregistered version of the resource-rational strategy selection model

The preregistered version of the resource-rational strategy selection model is a special case of the version described in the main text and does not include a trembling-hand error (i.e., ϵ is set to 0). The model-fitting procedure was as described in the main text, with the following exceptions: First, the grid search only involved two free parameters (strategy set S and cost-weighting parameter δ). Second, the possible values of S ranged from single-strategy sets to the set including all eleven strategies). Third, we selected the best-fitting parameter combination based on predictive accuracy for the empirically observed choices (instead of log-likelihood).

To examine to what extent the preregistered version of the resource-rational strategy selection model was able to capture the participants' choices, we conducted posterior predictive checks. For this purpose, we simulated choices of every participant in the experimental task 100 times, using the individual best-fitting parameter values. For every participant, we computed the proportion of trials in which the choice predicted by the model matched the empirically observed choice. In younger adults, the proportion of matching choices was, on average, 0.74 (range: 0.61–0.89) and in older adults 0.73 (range: 0.56–0.94). A two-sided Bayesian t-test indicated moderate evidence that the match of the simulated with the empirically observed choices did not differ

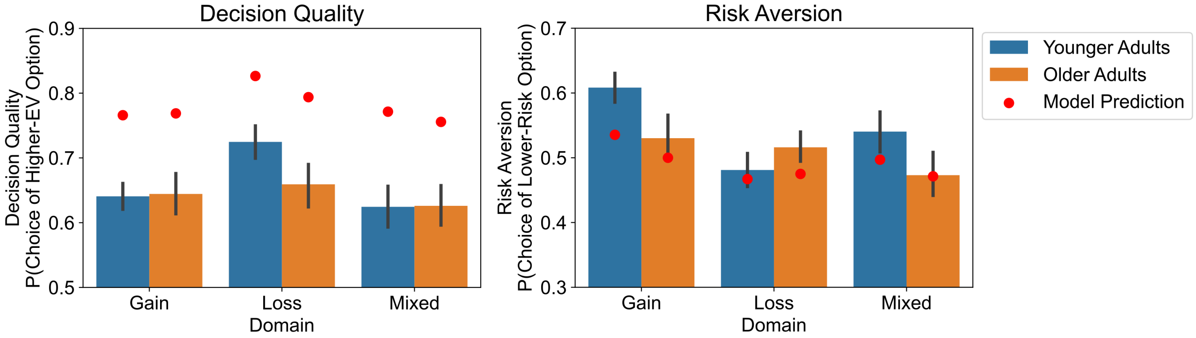

Figure S6.1

Decision quality (left) and risk aversion (right) by problem domain and age group. Bars show the empirically observed behavior (with error bars representing standard error of the mean), red dots show average predictions of the model simulations of the preregistered model version (without trembling-hand error).

between age groups ($BF_{10} = 0.32$). The higher accuracy of the model version without the trembling-hand error parameter is to be expected: Any model with a higher accuracy than chance will necessarily show a better fit when noise (i.e., unsystematic variability) is reduced—as is the case when the trembling-hand error is not included.

We also assessed how well the preregistered version of the resource-rational strategy selection model captured the empirically observed age differences in decision quality and risk aversion. Similar to the posterior predictive checks reported for the model version with a trembling-hand error, we analyzed the predicted decision quality and risk aversion of the fitted resource-rational strategy selection model (averaged across the 100 model simulations) with a hierarchical beta regression with age group (younger vs. older), problem domain (gain, loss, and mixed), and their interaction as fixed effects and random intercepts for participants and choice problems. In trials in which the model predicted the same choice across all 100 simulation runs, the values for the averaged decision quality or risk preference of either 0 or 1 were replaced with 0.001 and 0.999, respectively, thus ensuring that the data is suitable for the regression analysis. Mirroring the empirical findings, there was lower decision quality in loss problems for the simulated choices of the older adults than for the simulated choices of the younger adults, although the 95% credible interval did not exclude 0 ($b = -0.08, \text{CI} = [-0.22, 0.05], d = -0.10$; cf. dots in Figure S6.1). Furthermore, similar to the empirically observed age effects, the simulated choices of older adults showed lower risk aversion than those of younger adults in gain problems ($b = -0.09, \text{CI} = [-0.21, 0.04], d = -0.10$) and in mixed problems ($b = -0.06, \text{CI} = [-0.19, 0.06], d = -0.07$); again, the 95% credible intervals did not exclude 0. In conclusion, the version of the resource-rational strategy selection model without a trembling-hand error is able to capture the age differences in risky choice, but the replicated age effects are less pronounced than in the model version with a trembling-hand error.

Table S6.1

Tests of the hypotheses: Preregistered versus final version of the resource-rational strategy selection model.

Hypothesis	Model with trembling-hand error (reported in article)	Model without trembling-hand error (preregistered version)
Strategy-distribution hypothesis	$BF_{10} = 1.6 \times 10^{163}$	$BF_{10} = 6.7 \times 10^{179}$
Strategy-complexity hypothesis	$BF_{10} = 0.16$	$BF_{10} = 0.17$
Toolbox-size hypothesis	$BF_{10} = 0.98$	$BF_{10} = 0.61$
Strategy-selection hypothesis	$BF_{10} = 0.36$	$BF_{10} = 0.34$
Strategy-execution hypothesis	$BF_{10} = 0.55$	Not applicable
