# Supplementary material for: Older adults select different but not simpler strategies than younger adults in risky choice
Source: PLoS Comput Biol. 2024 Jun 10;20(6):e1012204. doi: 10.1371/journal.pcbi.1012204 (PMC11192436; doi:10.1371/journal.pcbi.1012204)
Supplement: S7 Text — (PDF) [file pcbi.1012204.s007.pdf]

Parameter recovery analysis

We assessed the identifiability of model parameters by simulating the choices of 1000 agents on the risky choice problems of Pachur, Mata, & Hertwig [1]. For every agent, a value of the cost-weighting parameter was drawn from a uniform distribution between 0 and 20 and value of the trembling-hand error parameter was drawn from a uniform distribution between 0 and 0.5. Every agent was equipped with a toolbox of k strategies, with $1 \leq k \leq 5$ and the distribution of k being balanced across all simulated agents, by randomly drawing k strategies from the set of all possible strategies without replacement. If one or more strategies out of an agent's toolbox were never selected on any choice problem, this agent was discarded and a new agent with an equally sized toolbox was simulated. The resource-rational strategy selection model was then fitted to these simulated choices.

For the cost-weighting parameter delta, the recovered parameter values were correlated with the data-generating parameters at $r = .48$. Because the cost-weighting parameter has no effect on choices for agents with only a single strategy in their toolbox (in this case, only one strategy can be selected, regardless of the weighting of costs), simulated agents with only one strategy in their toolbox were excluded from this analysis. For the trembling-hand error, the recovered parameter values were correlated with the data-generating parameter values at $r = .91$. The recovered toolbox sizes were correlated with the data-generating toolbox sizes at $r = .45$. When computing the average strategy cost for each agent, we found a correlation of $r = .52$ between the cost of the data-generating and the recovered strategies. On average, the data-generating strategy was recovered correctly in 60.1% of trials. For comparison, a model that guesses an agent's strategies would correctly recover the strategies in only 9.1% of trials.

References

[1] Pachur T, Mata R, Hertwig R. Who Dares, Who Errs? Disentangling Cognitive and Motivational Roots of Age Differences in Decisions under Risk. Psychological Science. 2017;28(4):504–518. doi:10.1177/0956797616687729.
