# Supplementary figures and images for: Older adults select different but not simpler strategies than younger adults in risky choice
Source: PLoS Comput Biol. 2024 Jun 10;20(6):e1012204. doi: 10.1371/journal.pcbi.1012204 (PMC11192436; doi:10.1371/journal.pcbi.1012204)

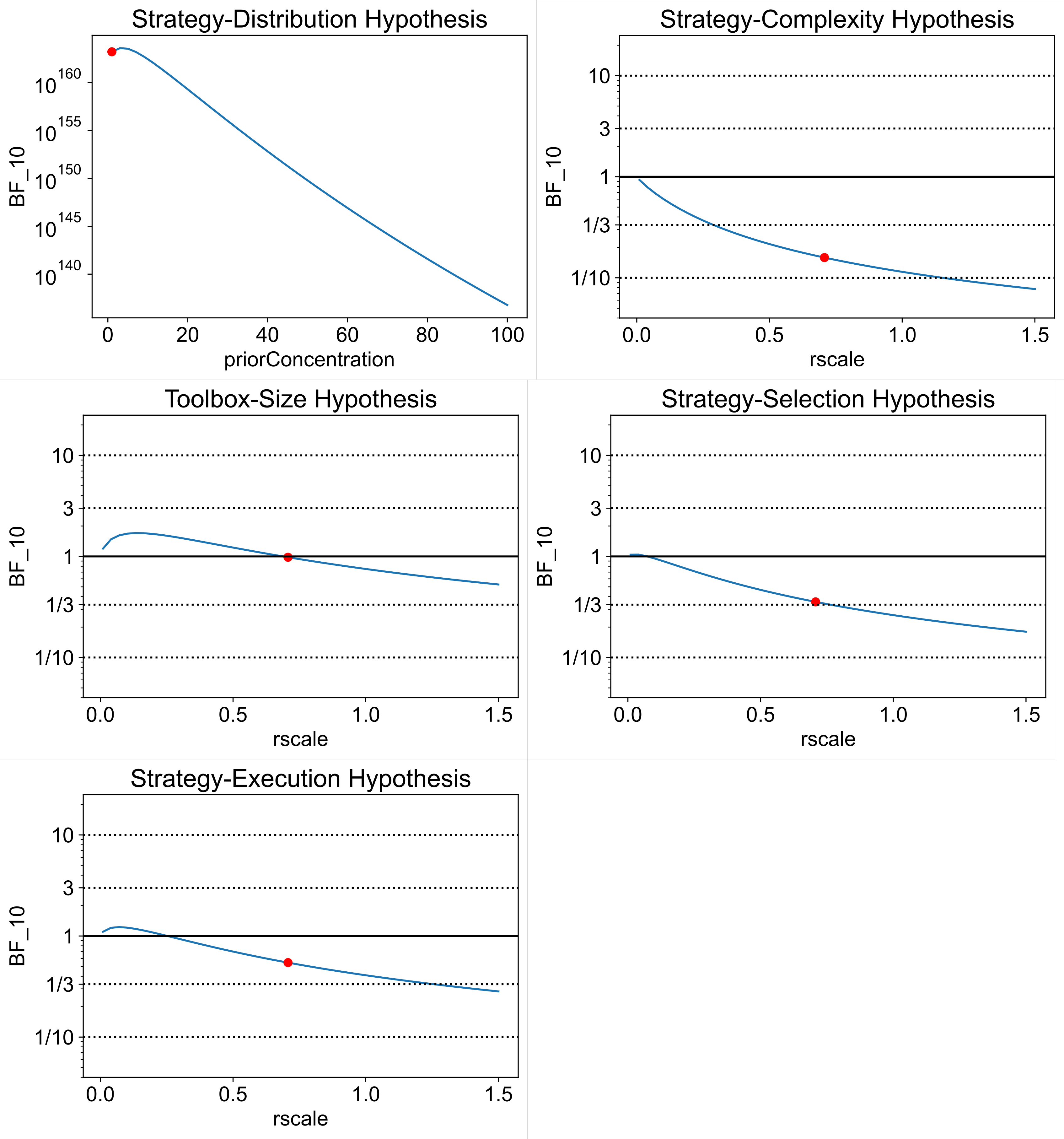

Supplement: S1 Fig — (TIFF) [file pcbi.1012204.s008.tiff]
