# Supplementary material for: Older adults select different but not simpler strategies than younger adults in risky choice
Source: PLoS Comput Biol. 2024 Jun 10;20(6):e1012204. doi: 10.1371/journal.pcbi.1012204 (PMC11192436; doi:10.1371/journal.pcbi.1012204)
Supplement: Attachment — Submitted filename: Rebuttal_Letter.pdf [file pcbi.1012204.s009.pdf]

Dear Dr. Palminteri,

Attached please find our revised manuscript entitled “Older Adults Select Different But Not Simpler Strategies Than Younger Adults in Risky Choice” (PCOMPBIOL-D-23-01819), which we would like to re-submit for publication in *PLOS Computational Biology*. We thank you and the two reviewers for their careful evaluation of our manuscript and the constructive comments.

Below we provide a detailed description of how we revised the manuscript in light of each comment. We would like to highlight and summarize our response to two major issues that were raised by you and the reviewers:

Model fit of the Resource-Rational Strategy Selection (RRSS) model
One important concern highlighted by Reviewer 2 and you was that the RRSS model may not provide a good fit to the empirical data. To address this concern, we now report as part of the posterior predictive checks an analysis of individual differences. This analysis demonstrates that the model captures between-subject variability in both decision quality and risk aversion rather well (R2.1). In addition, we compare the RRSS model to cumulative prospect theory (CPT) as a more established model of risky choice (R2.3). This analysis shows that cumulative prospect theory describes the choice behavior better than the resource-rational strategy selection model. However, we highlight that the key merit of the resource-rational strategy selection model is that, unlike cumulative prospect theory, it explicitly assumes the operation of cognitive processes underlying decision making–making the framework (unlike CPT) amenable to testing various ways in which age-related cognitive decline could manifest in the selection of cognitive strategies. In addition, we discuss possible reasons for why the RRSS model–although it generally captures age differences in risky choice–does not match the absolute level of participants’ decision quality and risk aversion perfectly (R1.4). Specifically, one important reason might be that the parameters of the RRSS model are assumed to be constant across problem domains (i.e., gains vs. loss vs. mixed problems), which is a simplifying assumption that is necessary to be able to reliably estimate the model parameters. (As an aside, it may be interesting to note that in the analyses of the dataset by Pachur et al. (2017), also CPT was not able to fit participants’ level of decision quality perfectly (see their Figure 1)–suggesting that this might be a more general modeling challenge.)

Generalization of the findings to another dataset
A question brought up by Reviewer 2 is the extent to which the findings of our modeling analyses may generalize. For this issue it is important to bear in mind that the key question underlying our current investigation is what conclusions about the specific dataset in Pachur et al. (2017), which has previously been examined with the formal framework of cumulative prospect theory (CPT), can be drawn when analyzed with an alternative framework that also allows to shed light on possible psychological mechanisms. In response to R2.2, we fitted the model to a second dataset of age differences in risky choice collected by Horn et al. (2023). To our knowledge, this is the only other dataset available with a sufficiently large number of choices per participant, which is necessary to estimate the RRSS model. Similar as for the Pachur et al. dataset, also this analysis for Horn et al. (2023) suggests that the two age groups do not differ in the complexity of the strategies used, the number of strategies available to them, the weighting of strategy costs, or strategy execution errors.

We are looking forward to hearing back from you.

Kind regards,

Florian Bolenz and Thorsten Pachur

Editor

As you can see the Reviewers expressed some serious concerns about the modelling results. In particular Reviewer 2 raises some important concerns about model validation that, if not addressed properly, will prevent the article being accepted. It is important to 1) show clear signatures of the models correctly fitting (or not) the data (see https://doi.org/10.1016/j.tics.2017.03.011) and 2) fit the model to all datasets. Of course, all the other issues raised by Reviewer 1 and Reviewer 2 have to be taken into account. Finally, please consider this https://psyarxiv.com/kyfus/download?format=pdf when preparing you rebuttal letter.

Please see our summary above.

Reviewer 1

Major Comments

R1.1

#1 Strategy overlap

From the current version of the paper, it is unclear what decisions are made overall by each of the strategies considered. Particularly, no detail is given about overlapping decision profiles between strategies. In other words, do the different strategies have clearly distinct decision patterns or is there a substantial overlap between them? Are some strategies indistinguishable in terms of decisions made in the set of problems? This is of prime importance considering that the model is fitted on the only binary choice made by subjects and that for each problem many strategies make the same decision. Could the authors provide details about the decision profile (across problems) of the strategies?

We thank the reviewer for raising this important issue. In response, we conducted the proposed analyses and report the extent to which the different strategies overlap in their choices in the current dataset in the revised manuscript. This analysis is reported in the Supplementary Information (section "Comparison of the Strategies' Decision Profiles"). In a nutshell, the analyses show that most strategies differ considerably in their decision profiles. On p. S8 we now write:

> "To analyze the extent to which the strategies considered in our analysis have distinguishable decision profiles, we simulated the decisions of each strategy on the risky choice problems of Pachur et al. (2017) 100 times. For each pair of strategies, we computed the percentage of problems for which both strategies predicted the same choice. For most of the strategies (8 out of 11), the overlap between their decision profiles was less than 77%. Three strategies (Most-likely, Probable, Lexicographic) showed highly similar decision profiles (more than 92% overlap). However, since in our modeling analysis none of these strategies was estimated to be selected frequently by participants of either age group, the effect of potential confusion between these three strategies should be negligible."

We point to this analysis when describing the computational model (Methods, subsection "Computational model") on p. 21:

> "In our analyses, we consider a comprehensive set of cognitive strategies previously suggested to be relevant for risky choice (Brandstätter et al., 2006; Thorngate, 1980, see Table 1 for a detailed description). *Most of these strategies have clearly different decision profiles for the choice problems in Pachur, Mata, and Hertwig (2017) (see Supplementary Information).*" (italics highlight added text)

R1.2

#2 "Strategy recovery"

This comment is related to the previous one and concerns the distinguishability of the

strategies considered, in term of decisions produced. In the supplementary materials is presented a parameter recovery analysis which show that model parameters can be fairly well recovered. What is not clear is wether strategies themselves can be recovered. If I understand well, for a given strategy set size k, a same delta value implies that the exact same strategies will be selected by the model for all problems. However, slightly different delta value could imply different strategy selections. Could authors give details about the strategy profiles obtained with the generating parameters and with the recovered ones? Can strategies be fairly well recovered?

We agree with the reviewer that the robustness of the estimated set of selected strategies is an important issue. In response, we added the respective analysis–showing that the strategies selected can be recovered well–to the section “Parameter recovery analysis” in the Supplementary Information (p. S12):

> “On average, the data-generating strategy was recovered correctly in 60.1% of trials. For comparison, a model that guesses an agent’s strategies would correctly recover the strategies in only 9.1% of trials.”

R1.3

#3 Model conceptually hard to support

The key assumption of the resource-rational strategy selection model is that decision makers select a strategy based on a tradeoff of cognitive costs and expected benefits for each problem faced. In other words, they must compute the cost and expected value of each strategy in their “toolbox” before selecting the best one considering their sensitivity to cognitive cost. With this in mind, it seems peculiar to assume an account for cognitive cost in the selection, as it appears to have already been paid when computing the costs and benefits of each strategy. Could the authors give more details about this aspect of the decision and the realism of this assumption?

Thanks for highlighting this, which is indeed a potentially confusing issue. To avoid confusion, we revised the manuscript to clarify the assumptions in the RRSS model (Lieder & Griffith, 2017) regarding the key variables guiding strategy selection: the expected cost and the expected value of a strategy in a given situation. Most generally, it is assumed in the RRSS model that the decision maker has learned (from feedback on previous occasions) an association between cues present in a given decision problem (e.g., distribution of probabilities, similarity of attributes across options) and the costs and accuracy expected for a given strategy. Importantly, however, this does not mean that the decision maker consciously and explicitly calculates the costs and accuracy during subsequent strategy selection. Rather, similar as in reinforcement learning it is assumed that an expected value for an option is learned from feedback, it is assumed that expected costs and payoff are mentally represented in terms of learned associations with the cues present in the decision problem. In our modeling analysis, we make the simplifying assumption that these represented quantities are approximated by the actual costs and actual payoff, but we do not explicitly model this approximation process (nor the foregoing learning process). We clarify these assumptions now when describing the computational model (Methods, subsection “Computational model”; p. 21f):

> “The theory of resource-rational strategy selection assumes that a strategy’s expected payoff $r_{s,p}$ and expected cost $c_{s,p}$ are approximated by internal predictive models based on the features of a choice problem (e.g., distribution of probabilities, similarity of attributes across options; Lieder & Griffiths, 2017). These predictive models could be acquired, for instance, via reinforcement learning and be mentally represented as the weights of the features in the predictive model. This predictive model allows the decision maker to approximate a strategy’s expected payoff and cost even without actually executing the strategy and also for new choice problems. In our modeling analysis, we make the simplifying assumption that the represented quantities are approximated by a given strategy’s actual cost and actual payoff, but we do not explicitly model this approximation process.”

R1.4

#4 Quality of fit

The model does not seem to be able to capture some parts of the data, particularly the choice quality of younger adults in losses and risk aversion of younger adults in gains. Could the authors explain why this is the case? What mechanisms or constraints prevent the model from accounting for these behaviours?

Although we can only speculate at this point, in our view one important constraint of the RRSS model as implemented here is that the parameters are not allowed to vary across choice domains (i.e., gain vs. loss vs. mixed). For example, allowing for differences in the weighting of strategy cost between choice domains could improve the fit of the model to behavioral differences between these domains. Given the amount of data required for model fitting, it is not feasible to estimate model parameters separately for the different choice domains in our analysis. Importantly, however, it is important to note that the model captures the qualitative differences between the choice domains even without assuming differences in the model parameters. We now address this issue more explicitly in the Discussion (p. 19):

> “The resource-rational strategy selection model is able to capture differences in the direction of the effect of age between gain, loss, and mixed problems without assuming parameter differences between domains. This suggests that the apparently complex interaction between age group and domain on risk aversion in the empirical data may be due to simple differences in strategy use—which in turn produce different risk propensities even if strategy selection is invariant across domains. *Still, allowing strategy selection to differ between domains might even further improve the fit of the model to the empirical data (e.g., aligning the model predictions more closely with the empirically observed behavior of younger adults with respect to decision quality in the loss domain and risk aversion in the gain domain). There is evidence that people invest more cognitive resources in problems involving losses (Yechiam & Hochman, 2013); they might therefore make different trade-offs between payoff and cost as a function of the problem domain. Estimating the model parameters reliably for each of the choice domains separately would, however, require more data per participant than are available in the dataset analyzed in the current study.*” (italics highlight newly added text)

As an aside, it may be interesting to note that also in the analyses of the dataset by Pachur et al. (2017), CPT was not able to match participants’ level of decision quality perfectly–see

their Figure 1. This suggests that the issue highlighted by the reviewer represents a more general modeling challenge that not only applies to the RRSS model.

Minor Comments

R1.5

#1 Strategy complexity check

In the discussion, authors emphasise the complexity of quantifying cognitive costs of the different strategies. Considering the method used here, is it possible to see in the data a relation between cognitive costs and strategy execution? Are most costly strategies more prone to execution error?

Thanks for this interesting comment. In our analysis, the trembling-hand error, which indexes error in strategy execution, is estimated for each participant but is assumed to be the same across all strategies (estimating a separate execution error for each strategy would require estimating 10 additional free parameters per participant, which is unfeasible for the given dataset). We nevertheless tested the reviewer's suggestion by comparing at each trial the consistency between the empirically observed choice and the predicted choice of the strategy estimated by the model. When regressing this measure of consistency at each trial on the cost of the model-predicted strategy with a mixed-effect logistic regression analysis, there was actually a *negative* relationship between consistency and strategy costs–that is, the probability of a participant deviating from a strategy was lower for strategies with higher costs (b = -0.10, p = 0.002). Additional analyses showed that this effect is fully mediated by option similarity (operationalized in terms of how close the expected values of the two risky gambles are). Specifically, the costs of the predicted strategy are lower for choice problems in which the options have similar expected values than for choice problems in which the options have rather different expected values (b = 0.16, p < 0.001). This naturally follows from the resource-rational framework underlying our model: when both options are very similar in value, the expected payoff of all strategies in an agent's toolbox is almost identical; in this case, the agent should select a low-cost strategy. When including option similarity as a predictor in the logistic regression, the effect of strategy cost on execution error was no longer significant (b = -0.06, p = 0.06). In summary, this suggests that the correct application of a strategy does not differ as a function of the cost of the strategy.

Reviewer 2
Major comments

R2.1
A primary concern is that the resource-rational strategy selection model used fit to the data does not provide strong evidence to support the author's claims. The model simulations mainly predicted age-invariant results but were not well-fit to the age-related differences in decision quality and risk aversion (e.g., in Figure 1). Additionally, given that the model revealed no significant age differences in key model parameters (e.g., strategy cost, toolbox size, cost-weighting parameters, error), the model results are rather inconclusive and difficult to interpret in terms of how they relate to cognitive (or motivational) differences between age groups.

Thanks for the comment. We would like to clarify that the model does not mainly predict age-invariant results. Instead and importantly, the model actually reproduces the age differences in risk aversion (gain problems: b = -0.12, CI = [-0.20, -0.03], d = -0.10; mixed problems: (b = -0.12, CI = [-0.21, -0.03], d = -0.10) and (to a lesser extent) in decision quality (loss problem: b = -0.05, CI = [-0.19, 0.09], d = -0.05). Further, we would like to clarify that although it is correct that the results show no differences in the cost-weighting parameter, the trembling-hand error, strategy cost, and toolbox size, the model *does* show age differences in another important parameter estimated by the model, namely the composition of the toolbox of strategies–see Figure 3. This is represented by the free model parameter *S* (which then translates into the different patterns of selected strategies). As our analyses show, these differences in estimated toolbox composition capture the age differences in risk aversion rather well. To avoid misunderstandings, we highlight this important insight about age differences from our modeling analyses now in the Discussion (p. 17):

> “Instead, the age differences in decision making resulted from different configurations of participants’ strategy toolboxes.”

Second, to demonstrate that the model captures individual differences, we compared the empirical choices and the model predictions for each individual participant with respect to decision quality and risk aversion. This analysis shows high correlations between empirical behavior and model predictions. This is reported in the Results section (p. 12):

> “Additionally, we compared for each participant their empirically observed decision quality and risk aversion (pooled across all three problem domains) against the decision quality and risk aversion of the choices simulated with their best-fitting model parameters (Figure 2). The higher a participant’s decision quality, the higher was the decision quality of their simulated choices ($r = 0.91, BF_{10} = 5.7 \times 10^{41}$). Moreover, the higher a participant’s risk aversion, the higher was their simulated risk aversion ($r = 0.79, BF_{10} = 3.1 \times 10^{23}$). This analysis shows that the resource-rational strategy selection model captured the individual differences between participants in choice behavior well.”

Overall, these analyses underscore that the estimated RRSS model captures many details of the empirical data, both on the aggregate level and the individual level.

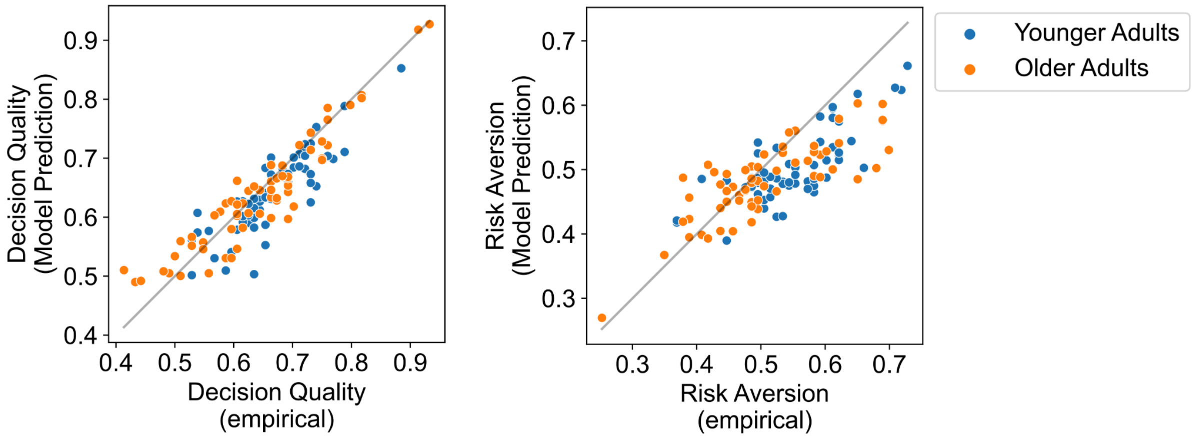

Figure R1 (Figure 2). Comparison of simulated (based on the fitted resource-rational strategy selection model) and empirically observed levels of decision quality (left) and risk aversion (right). Each circle represents one participant (with the choices pooled across the three domains; i.e., gain, loss, and mixed). The diagonal lines indicate identity (i.e., perfect fit).

R2.2

The model was only fit to one dataset, which limits the generalization of these findings. It would be helpful to replicate these modeling results in a different dataset with a similar task, which could provide more compelling evidence for model validation.

We share the reviewer's view that replicating our analysis on a different dataset would further strengthen our conclusions. Note that the motivation for the current analyses was to see how the differences between younger and older adults in risk aversion and decision quality observed in the dataset by Pachur et al. (2017)--which has previously only been scrutinized with cumulative prospect theory (CPT)--would be reflected when analyzed with a computational modeling framework that allows more cognitive insights than CPT (which is mute with regard to cognitive processing). Therefore, generalizing our modeling conclusions to another dataset requires a dataset that is not only based on a similar set of lottery problems and shows the same behavioral patterns, but also allows for a rigorous computational modeling analysis (in terms of a sufficient amount of individual-level data points). In the dataset by Pachur et al. (2017), to which we apply the RRSS model, each of a total of 122 participants was presented with 105 choice problems.

To our knowledge, the only other currently existing dataset on risky choice and aging that involves a comparable amount of data is a recent study by Horn et al. (2023), where each participant was presented with 78 choice problems. One important aspect of this dataset, however, is that although the qualitative patterns of the age differences in decision quality and risk aversion across the different domains (i.e., gain, loss, and mixed) were exactly the same as in Pachur et al. (2017)--see Figure R2 below–these differences were smaller and not statistically significant. One possible reason is that the data by Horn et al. (2023) were collected via MTurk, where age effects can be considerably attenuated relative to data

collected in the lab–for instance, because older participants that are recruited on MTurk tend to be highly educated (see Greene & Naveh-Benjamin, 2022, Psychology and Aging). Indeed, whereas in Pachur, Mata, and Hertwig (2017) about 45% of the older adults had a university degree (see their Table 1), this was the case for about 65% of the older adults in Horn et al. (2023; see their Table 1). (For the younger adults, by contrast, the proportions were similar in the two datasets, with 40% and 47%, respectively, of participants with a university degree.) Despite the similarities in methodology, the datasets of Pachur, Mata, and Hertwig (2017) and of Horn et al. (2023) are thus not directly comparable.

With that complication in mind, we nevertheless fitted the resource-rational strategy selection model to the Horn et al. (2023) dataset to test whether also here the RRSS model would fit the data and suggest no differences between younger and older adults in cognitive aspects of strategy selection (although the sample of older adults in Horn et al., 2023, had higher education levels than that in Pachur et al., (2017), they still had lower measures of fluid abilities than the younger adults--but the effect size of this age difference was smaller than in Pachur et al., 2017: Cohen's d = 1.22 vs. 2). Note that younger and older adults may achieve similar decision quality and risk aversion using different strategies (e.g., Li, Baldassi, Johnson, & Weber, 20213, Psychology and Aging). In a nutshell, this analysis showed similar patterns as for the Pachur et al., 2017, data: Again, there were no differences between older and younger adults in toolbox size, strategy complexity, weighting of strategy cost, and execution error. In addition, the RRSS again captured key aspects of the data rather well (see Figure R2). Finally, despite younger and older adults showing no differences in decision quality and risk aversion, the distributions of the strategies that the RRSS estimated to be used by younger and older adults differed somewhat. These age differences in strategy use were considerably less pronounced than in the Pachur et al. (2017) data (Cramer's V = 0.11 vs. 0.25). This means that younger and older adults seem to achieve the same levels of decision quality and risk aversion, but use a somewhat different set of strategies.

We report the analysis of the Horn et al. (2023) dataset–including behavioral analyses, modeling with the RRSS, a model comparison with CPT, and a model recovery analysis–in the Supplementary Information (p. 14ff):

> "To examine the generalizability of our conclusions for the dataset by Pachur et al. (2017), we applied the resource-rational strategy selection model to the dataset collected by Horn et al. (2023), who also examined risky choices in younger and older adults. To our knowledge, this is the only other dataset available for which there is a relatively large number of choices per participant, which is a prerequisite for reliably estimating the model parameters. It should be noted, however, that Horn et al. (2023) did not find age differences in decision quality and risk aversion (a likely reason for this discrepancy with regard to the findings by Pachur et al. (2017) is that their sample of older adults had considerably higher education levels). In that sense, the datasets by (Horn et al., 2023) and (Pachur et al., 2017) are somewhat difficult to compare. Still, given that similar decision behavior might be achieved by younger and older adults using different strategies (e.g., Li et al., 2013), the dataset by Horn et al. (2023) might still be an informative testbed for the current purposes.

Horn et al. (2023) had collected risky choices from 147 younger adults (71 female, 68 male, 8 other, mean age: 25.4 years, range: 18–36 years) and 139 older adults (79 female, 60 male, mean age: 70.2 years, range: 60–89 years), recruited via Prolific Academic. Participants completed 78 risky choice problems, consisting of two gambles each with two outcomes and corresponding probabilities. Choice problems were evenly distributed across the gain, loss, and mixed domain. The type of incentivization was manipulated between participants; half of the participants received a financial bonus based on one randomly drawn choice and the other half received a financial bonus based on all their choices. Because there were no differences in decision quality and risk aversion between incentivization conditions, we collapsed both conditions for the purpose of our current analysis.

Posterior predictive checks

For each participant, we computed the proportion of trials in which the choice predicted by the model matched the empirically observed choice (averaging across 100 model simulations). The proportion of matching choices was, on average, 0.70 in younger adults (range: 0.50–0.95) and 0.67 in older adults (range: 0.51–0.84). A two-sided Bayesian t-test indicated moderate evidence that the match of the simulated with the empirical choices differed between age groups (BF_{10} = 3.6). In other words, the resource-rational strategy selection model seemed to capture the choices of younger adults slightly better than those of older adults.

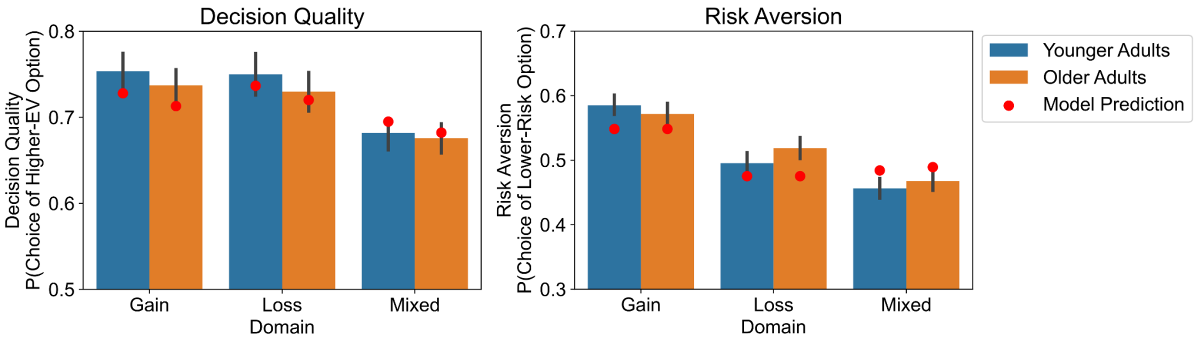

Figure R2 (Figure S4). Decision quality (left) and risk aversion (right) by problem domain and age group in the dataset by Horn et al. (2023). Bars show the empirically observed behavior (error bars represent the standard error of the mean), red dots show the average predictions of the resource-rational strategy selection model.

Figure S4 shows the decision quality and risk aversion as empirically observed and as predicted by the resource-rational strategy selection model. Since in the Horn et al. (2023) dataset the age differences in decision quality and risk aversion were not statistically significant, we did not conduct a statistical analysis of age differences in the model predictions. Note, however, that the model described the overall behavioral patterns in the data quite well.

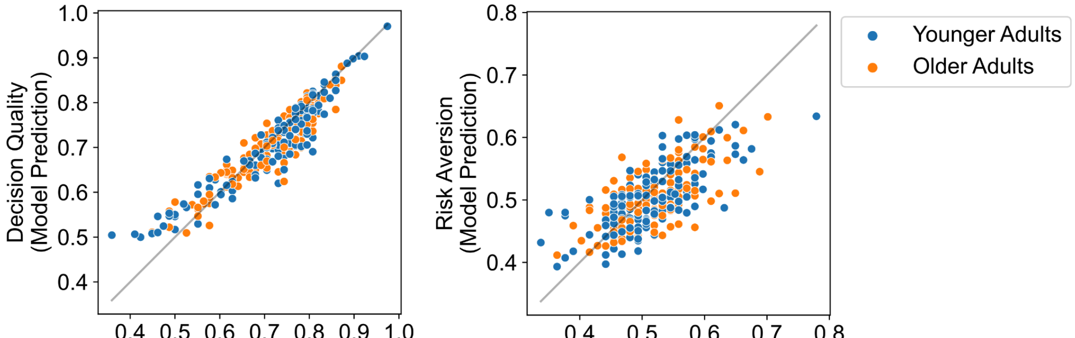

Figure R3 (Figure S5). Comparison of simulated (based on the fitted resource-rational strategy selection model) and empirically observed levels of decision quality (left) and risk aversion (right) in the dataset by Horn et al. (2023). Each circle represents one participant (with the choices pooled across the three domains; i.e., gain, loss, mixed). The diagonal lines indicate identity (i.e., perfect fit).

We also compared for each participant their empirically observed decision quality and risk aversion (pooled across all three problem domains) against the decision quality and risk aversion of the choices simulated with their best-fitting model parameters (Figure S5). The higher a participant's decision quality, the higher was the decision quality of their simulated choices ($r = 0.94, BF_{10} = 1.0 \times 10^{127}$). Moreover, the simulated levels of risk aversion were strongly correlated with the empirical ones ($r = 0.69, BF_{10} = 6.1 \times 10^{37}$). This analysis shows that similar as for the data by Pachur et al. (2017) reported in the main text, the resource-rational strategy selection model captured the individual differences in choice behavior between participants rather well.

Age differences in model parameters

Although younger and older adults did not differ in decision quality and risk aversion, they might still rely on different strategies. A Bayesian contingency-table test showed that there was strong evidence that the distribution of strategies differed between age groups ($BF_{10} = 2.8 \times 10^{47}$, Cramér's V = .11; Figure S6). The strategies with the more pronounced age differences in strategy use were the least-likely strategy (more often selected by older adults) and the equal-weight strategy (more often selected by younger adults).

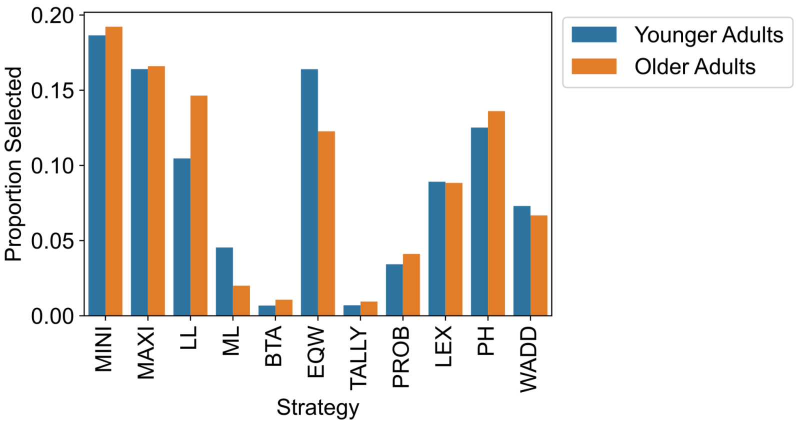

Figure R4 (Figure S6). Frequency of strategies across all trials in the dataset by Horn et al. (2023). MINI: Minimax, MAXI: Maximax, LL: Least-likely, ML: Most-likely, BTA: Better-than-average, EQW: Equal-weight, TALLY: Tallying, PROB: Probable, LEX: Lexicographic, PH: Priority heuristic, WADD: Weighted-additive.

To compare model parameters between younger and older adults, we conducted one-sided Bayesian t-tests. There was moderate evidence that older adults did not incur lower costs by the strategies used ($BF_{10} = 0.11$), moderate evidence that older adults did not have smaller strategy toolboxes ($BF_{10} = 0.19$), strong evidence that the cost-weighting parameter was not larger for older adults ($BF_{10} = 0.07$), and inconclusive evidence that the trembling-hand error is higher for older adults (BF_{10} =

2.3). Overall, these findings corroborate the conclusion from the main text that age differences in strategy selection are not predominantly driven by cognitive factors.

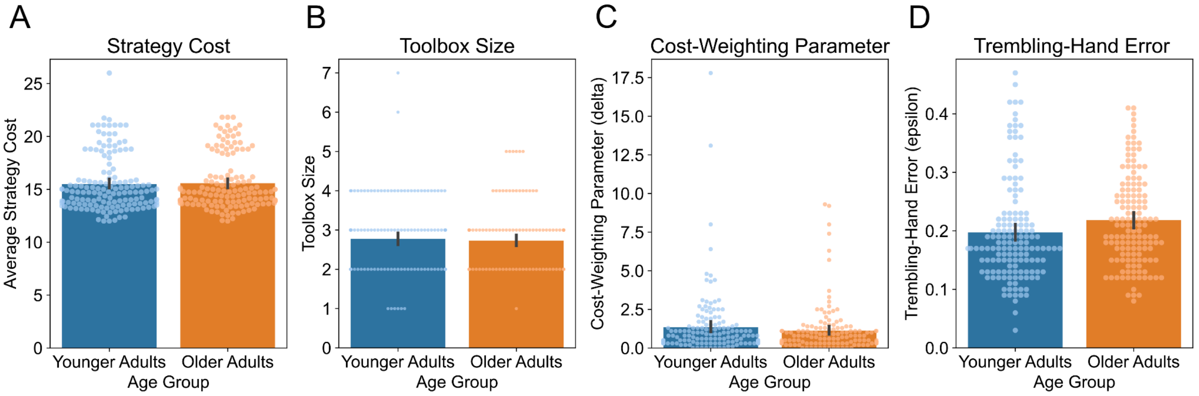

Figure R5 (Figure S7). Average strategy cost (A), toolbox size (B), cost-weighting parameter (C), and trembling-hand error (D) for younger and older adults in the dataset by Horn et al. (2023). Bars indicate group means, with error bars representing the standard error of the mean. Points represent individual participants.

Parameter recovery analysis

We conducted a parameter recovery analysis similar to the analysis conducted for the choice problems from Pachur et al. (2017) also for the Horn et al. (2023) data. For the cost-weighting parameter delta, the recovered parameter values were correlated with the data-generating parameters at r = .35. For the trembling-hand error, the recovered parameter values were correlated with the data-generating parameter values at r = .90. The recovered toolbox sizes were correlated with the data-generating toolbox sizes at r = .50. When computing the average strategy cost for each agent, we found a correlation of r = .47 between the cost of the data-generating and the recovered strategies. On average, the data-generating strategy was recovered correctly in 53.7% of trials. For comparison, a model that guesses an agent’s strategies would correctly recover the strategies in only 9.1% of trials.

Model comparison

When comparing the model fit between the resource-rational strategy selection model and cumulative prospect theory, cumulative prospect theory captured the choice data better than the resource-rational strategy selection model for both younger and older adults (Table S3).

Model	Full sample	Younger adults	Older adults
Resource-rational strategy selection	-10926	-5434	-5492
Cumulative prospect theory	-10712	-5330	-5382

Table R1 (Table S3). Log-likelihoods of the resource-rational strategy selection model and cumulative prospect theory for the dataset by Horn et al. (2023). Higher values indicate a better fit.

R2.3
It would also be helpful to compare the RR model to other extant models, which would demonstrate this model performs better than alternate models of risky DM.

We appreciate this comment. Please note that the key motivation and goal of the current analyses was to take an explicitly different perspective than existing models that are commonly used to model age differences in risky choice–in particular CPT and expected utility (EU) models, which are mute with regard to the cognitive processes underlying people's choices. Therefore, a key merit of the RRSS model is that it allows for insights that go beyond those that can be obtained with EU-based models such as CPT (of course, as long as RRSS is generally able to capture the data). Therefore, a model comparison of the RRSS with CPT (which is nested under the EU theory but typically outperforms it) is of limited value for the present purposes.

Nevertheless, in order to give the reader a general sense of model fit, we added an analysis comparing the resource-rational strategy selection and cumulative prospect theory in terms of log-likelihood. The results showed that the choices were better captured by cumulative prospect theory. Note that our model comparison does not take into account differences in model complexity because the number of free parameters in the RRSS is not clearly defined due to the categorical nature of the strategy toolbox parameter *S* (with more than 3,000 different levels). Also, leave-one-out cross validation is not feasible due to the long duration of model fitting (around 6h per participant on a HPC cluster with 32 parallel cores). We report the model comparison in the Results section (p. 12f):

> "We also compared the resource-rational strategy selection model to cumulative prospect theory (Tversky & Kahneman, 1992), arguably the most prominent model for risky choice. Cumulative prospect theory showed a better fit for the choice data in terms of higher log-likelihood than the resource-rational strategy selection model (Table 2). While this pattern was evident in both age groups, it was more pronounced for the younger adults than for the older adults. This analysis does take into account differences in model complexity because it is not straightforward to quantify the number of free parameters of the resource-rational strategy selection model due to the categorical, high-dimensional nature of the strategy toolbox parameter. With that limitation in mind, cumulative prospect theory describes the choice data better than the resource-rational strategy selection model."

Log-likelihood of the resource-rational strategy selection model and cumulative prospect theory.

Model	Full sample	Younger adults	Older adults
Resource-rational strategy selection	-7236	-3544	-3692
Cumulative prospect theory	-6666	-3025	-3640

Table R2 (Table 2). Log-likelihood of the resource-rational strategy selection model and cumulative prospect theory

We also discuss this finding in the Discussion (p. 18f):

> "The computational framework of resource-rational strategy selection offers a new perspective on age differences in risky choice. In contrast to approaches commonly used to model age differences in risky choice, such as expected utility theory (e.g., Tymula et al., 2013) or cumulative prospect theory (e.g., Horn et al., 2023; Pachur, Mata, & Hertwig, 2017), findings from the resource-rational strategy selection model are directly interpretable in terms of cognitive information processing. *Therefore, the resource-rational strategy selection model is able to provide novel insights into the psychological processes underlying age differences in risky choice even though in its current formalization it does not capture the choice behavior as well as models based on expected utility theory. As demonstrated with our current analyses, the resource-rational strategy selection model allows one to derive and test various hypotheses on different ways in which cognitive decline might affect cognitive processing. Further, being based on models of cognitive strategies, the resource-rational strategy selection model led to the observation that compared to younger adults, older adults seem to be less likely to process both outcome and probability information.*" (italics highlight newly added text)

Note that also in the analysis of the dataset by Horn et al. (2023), cumulative prospect theory has a higher log-likelihood (i.e., better fit) than the resource-rational strategy selection model (see Table R1).

R2.4

The authors argue that age-related differences are motivational in nature, but there is no evidence to support this claim. Given that the RR model dissects cognitive strategies, and that there is no explicit link to motivational factors (e.g., self-report, model parameters that reflect affective or motivational measures), these claims are not substantiated. Moreover, it is not clear whether OA considered outcomes (and not probability) due to capacity limitations (e.g., OA may only be able to process one dimension at a time vs. YA and can integrate both dimensions of outcome and probability more easily) or even attentional differences (e.g., OA prioritize outcome information vs. YA prioritize both). Overall, greater work is needed to

demonstrate why these age-related differences relate to psychological factors involved in risky decision-making, thus limiting the impact of the current results.

Thanks for highlighting this issue. We very much agree that our analysis does not establish a direct link between motivational factors and the observed age differences in risk taking. The modeling analysis shows that the age differences do not necessarily need to be understood as a consequence of cognitive decline, as we did not observe age differences in the model parameters that reflect cognitive resources (e.g., the cost-weighting parameter or the overall complexity of strategies). As one possible alternative reason for the observed age differences in strategy selection, we consider motivational factors. Specifically, older adults typically report less negative and more positive affect than younger adults, and Pachur, Mata, and Hertwig (2017) showed that these age differences in affect mediated age differences in risk aversion. (In addition, note that negative and positive affect have been shown to be negatively and positively, respectively, associated with willingness to take risks; e.g., Lerner & Keltner, 2001). We would like to emphasize, however, that such a motivational perspective is only *one* possible explanation for the age differences in strategy selection suggested by our current analysis. We bring up the issue of motivation primarily to have a basis for a constructive discussion of our results (which might be taken up in future research). To avoid misunderstandings, we make our argument clearer in the Abstract (p. 2):

> "Our modeling approach suggests that age differences in risky choice are not necessarily a consequence of cognitive decline; instead, they may reflect motivational differences between age groups."

In further response to the reviewer's comment, we conducted and now report a mediation analysis that quantifies the extent to which age differences in risk aversion (as they are captured by the fitted RRSS model) are accounted for by individual differences in positive and negative affect (which were also measured by Pachur et al., 2017), a central facet of motivation. This analysis shows that around 30% of the age effect is mediated by age differences in positive affect and negative affect. The mediation analysis is reported in the Supplementary Information (p. S10f):

> "To further examine the viability of a motivational account of age differences in risky choice, we assessed to what extent the effect of age was mediated by differences in positive and negative affect. In the study by Pachur et al. (2017), positive and negative affect were assessed with a short version of the Positive Affect Negative Affect Schedule–Extended (PANAS-X; Grühn et al., 2010). Two older adults, for whom these variables were not recorded, were not included in this analysis. As reported in Pachur et al. (2017), the older adults reported lower negative affect and higher positive affect than the younger adults (which is a typical finding, see Grühn et al. (2010) and Kunzmann et al. (2000)).
>
> To compute the total effect of age group on model-predicted risk aversion, we used mixed-effects beta regression to predict the proportion of choices of the option with the lower risk according to the simulations of the fitted resource-rational strategy selection model, using age group (younger vs. older), problem domain (gain, loss, and mixed), and their interaction as fixed effects, and with random intercepts for participants and choice problems. Except from the slightly lower sample size, this

analysis was identical to the posterior predictive check reported in the main text. To compute the direct (i.e., unmediated) effect of age group on model-predicted risk aversion, we fitted an extended regression model that additionally included positive affect, the interaction of positive affect and problem domain, negative affect, and the interaction of negative affect and problem domain. The indirect (i.e., mediated) effect of age group on model-predicted risk aversion was computed by subtracting the direct effect from the total effect. To quantify the strength of the mediation, we computed the ratio of the indirect effect over the total effect P_M (Shrout & Bolger, 2002).

Table S2 shows the total, direct, and indirect effect of age groups as well as the effects of positive affect and negative affect on model-predicted risk aversion, separately for gain and loss problems (the two domains for which age differences were observed). Although the credible intervals for the indirect effects did not exclude 0, the P_M ratios suggest that up to a third of the effect of age group on model-predicted risk aversion was mediated by positive and negative affect (gain problems: P_M = 0.33, mixed problems: P_M = 0.31)."

	Gain problems		Mixed problems	
Effect	*b*	95% CI	*b*	95% CI
Age group (total effect)	−0.12	[−0.20, −0.03]	−0.12	[−0.21, −0.03]
Age group (direct effect)	−0.08	[−0.18, 0.02]	−0.08	[−0.18, 0.02]
Age group (indirect effect)	−0.04	[−0.17, 0.10]	−0.04	[−0.17, 0.10]
Positive affect	−0.01	[−0.06, 0.04]	−0.02	[−0.07, 0.04]
Negative affect	0.04	[−0.02, 0.10]	0.04	[−0.02, 0.10]

Table R3 (Table S2). Mediation analysis testing to what extent the age differences in risk aversion as modeled with the resource-rationality strategy selection model are mediated by positive and negative affect.

In addition, we revised and clarified the respective paragraphs in the Discussion (see p. 17)

"*If the age differences in strategy use are not due to cognitive factors, what else might drive them? One possibility is that they reflect motivational differences.* Older adults typically report more positive affect than younger adults, as observed in Pachur, Mata, and Hertwig (2017), and more positive affect has been associated with lower risk aversion (Lerner & Keltner, 2001). *A mediation analysis reported in the Supplementary Information showed that around 30% of the age effect on risk aversion captured by the differences in strategy selection can be attributed to age differences in positive and negative affect—consistent with a motivational account of the age differences in strategy selection.*" (italics highlight newly added text)

Moreover, we agree with the reviewer that the framework of elementary information processes that we have used for quantifying strategy cost does not cover all conceivable aspects of strategy costs. We now address this more explicitly in our discussion of the limitations of this approach (p. 19f):

> “While this approach provides a useful proxy for strategy cost, more complex aspects of strategy cost are conceivable (e.g., differences in the costs of processing probabilities vs. outcomes; Peters & Bjalkebring, 2015), and quantifying cost more precisely remains an important task for future research (Fechner et al., 2019; Fechner et al., 2018; Thomson & Oppenheimer, 2022).”
